# Prevalence and Stigma of Postpartum Common Mental Disorders in the Gurage Region of Ethiopia: A Mixed-Methods Observational Cohort Study

**DOI:** 10.3389/fpsyg.2021.626797

**Published:** 2021-04-09

**Authors:** Sophia Monaghan, Meseret Ayalew Akale, Bete Demeke, Gary L. Darmstadt

**Affiliations:** ^1^Stanford University, Stanford, CA, United States; ^2^Project Mercy, Yetebon, Ethiopia; ^3^Department of Pediatrics, Stanford University School of Medicine, Stanford, CA, United States

**Keywords:** healthcare, postpartum mood disorder, stigma, mental health, maternal health, global health, global mental health

## Abstract

**Objectives:** Mental disorders are vastly underdiagnosed in low-income countries that disproportionately affect women. We aimed to evaluate the prevalence of common mental disorders in newly postpartum women, and stigma associated with mental health reporting in an Ethiopian community using a validated World Health Organization survey.

**Methods:** The Self Reporting Questionnaire (SRQ) for psychological distress was administered in Amharic by nurses to 118 women aged 18–37 years who had given birth in the prior 3 months in the Glenn C. Olsen Memorial Primary Hospital in Yetebon. Mental health stigma among the four nursing staff was assessed using Link and Phelan's Components of Stigma.

**Results:** Among 118 women surveyed, 18% had a probable common mental disorder using the SRQ 4/5 cutoff and 2% admitted to suicidal thoughts. Presence of stigma in the healthcare staff was verified, including labeling, stereotyping, separating, and status loss and discrimination.

**Conclusion:** Postpartum mental health disorders as well as stigma against such diagnoses are common in the Yetebon community. There is an urgent need for increased availability of properly trained and supervised healthcare staff in the identification and referral of postpartum women with common mental disorders.

## Introduction

Identification and treatment of mental illness in most African countries, including Ethiopia, remains scarce. The World Health Organization (WHO) identifies gender as a critical determinant of mental illness (World Health Organization, 2013), considering that unipolar depression (as opposed to bipolar depression) is twice as prevalent in women compared to men (Weber et al., 2019). Moreover, an estimated 20% of postpartum women in low-income countries suffer from common mental disorders, including depression and anxiety; a systematic review confirmed the prevalence of perinatal common mental disorders in Africa to be 18–19% (Sawyer et al., 2010). In addition to adverse reproductive health outcomes, risk factors for postpartum common mental disorders include a number of social dimensions such as being unmarried, marital conflict, family conflict, poverty, and lack of social support (Sawyer et al., 2010; Fisher et al., 2012).

The United Nations adopted global Sustainable Development Goals in 2015 that for the first time include a commitment (Target 3.4) to “by 2030, reduce by one-third premature mortality from non-communicable diseases through prevention and treatment and promote mental health and well-being” (UN General Assembly, 2015). The WHO has intensified its focus on mental health, calling, for example, for an end to the chaining of people with mental illness (WHO, 2011; Jack et al., 2014).

Attention to mental health is rising in Ethiopia (Hanlon et al., 2010; Ross et al., 2010). Ethiopia's health extension workers have received training on how to screen people for mental health problems and refer them for services, and are tasked with mental health prevention, promotion, and ongoing community-based care (Federal Democratic Republic of Ethiopia, Ministry of Health (MOH), 2012). Estimates for postpartum common mental disorders (e.g., depression, anxiety) in Ethiopia have ranged from 9 to 33% (Baumgartner et al., 2014). However, additional data is needed to refine estimates and to further inform policies on mental health services.

Mental health stigma appears to be prevalent in the Ethiopian culture, and may be inhibiting mental health recognition and treatment (Monteiro and Balogun, 2013b). Many in Ethiopia—more than half (56.5%) of study participants in Mekelle city in Northern Ethiopia—fail to recognize depression as a mental illness (Abbay et al., 2018). A number of studies have been conducted regarding the stoicism of the Ethiopian culture as well as the reluctance to show negative emotions, which may be a reason for lack of consistent discussion about emotions that may indicate mental illness (Neuner et al., 2012; Ayers et al., 2017; Evason, 2018). For example, a study conducted among mothers in Southern Ethiopia showed that their perception of infant emotions like fussing and crying were the result of physical illness as opposed to emotional upset (Bader and Fouts, 2018). Moreover, in the Southwest region of Ethiopia residents were likely to “deny those with mental illness their individual rights, prevent them from taking on various responsibilities and forbid people from marrying and living together” with those affected (Baumgartner et al., 2014). Those who suffer from depression or more serious psychotic illnesses may be considered to be weak or possessed by a demon (Reta et al., 2016) and are often marginalized and given unfair treatment in their communities. Such treatment may include social isolation, employment discrimination, and human rights violations. There has also been research on stigma within healthcare staff in particular. In a study of 61 postpartum nurses, an increase in stigma was associated with an increase in negative attitudes toward parenthood, a decrease in nursing interventions, and a lack of nursing support for mother's empowerment (Ordan et al., 2018). Therefore, it is important to understand if stigma is present in healthcare staff, and if so, whether it is affecting the proper diagnosis and treatment of women with postpartum mental disorders.

Our hypothesis is that postpartum mental disorders are more prevalent than reported previously in this region of Ethiopia, and that stigma in the healthcare staff may be an important factor in under-reporting. This study was conducted in order to address the lack of existing data on postpartum mood disorders and gain insight into the potential influence of stigma on ascertainment of mental health. Additionally, our consistent access to postpartum women from the community population during postnatal follow-up visits made this research feasible. The objectives of this research were: (1) to understand the prevalence of postpartum common mental disorders in a rural southern Ethiopian community, and (2) to assess whether stigma existed in the healthcare staff, potentially acting as one of the factors influencing the identification and treatment of mental illness.

## Methods

### Participants and Procedure

The study was conducted at Project Mercy, a holistic community development organization founded in 1977, which implements integrated programs in K-12 education, healthcare, food security, orphan care, adult skills and literacy enhancement, and infrastructure development. About 11,000 individuals are served in the 52-bed Glenn C. Olsen Memorial Primary Hospital each year.

Data were collected over a 9-month period, from October 2017 to June 2018. All women coming in for postnatal vaccinations and check-ups within 3 months of giving birth and providing verbal informed consent to participate in the survey were included. This was a time-limited, mixed-method, observational cohort survey. Among the healthcare staff who were being observed for stigma, few to no nurses in the Hospital had received any formal mental health pre-service education or in-service training.

### Sociodemographic Information

At the beginning of each survey, basic sociodemographic information was collected from the respondents including marital status, age, and number of children. This data was then housed in a separate document that was linked to the survey results through a number that was only available to SM and was kept in a password protected folder to ensure the survey results could not be linked back to the individual.

### Assessment of Postpartum Common Mental Disorders

Common mental disorders were assessed using the Self Reporting Questionnaire (SRQ) created by the WHO for use in primary health care settings in low- and middle-income countries (LMICs). The SRQ is a 20-question survey that asks yes or no questions and is scored based on the number of yes answers collected, with a scoring scale from 0 to 20 and a cut-off score of 4/5. The WHO uses the SRQ to assess the possible existence of non-specific psychological distress, including depression and anxiety disorders, as well as suicidality, in women in low-income countries, with further follow-up psychiatric assessment recommended to determine needed treatment (Beusenberg et al., 1994). The SRQ has been validated in neighboring Butajira, Ethiopia (Hanlon et al., 2008). Hanlon et al. provided the Amharic version of the survey, and a Project Mercy staff member made slight translation modifications, with attention to the most culturally appropriate way to word the questions to account for the Guragina language spoken in the population. The Amharic version of the survey was used in the interviews, however SM followed along on the back-translated English version to ensure fidelity in meaning of the questions.

The four nurses who rotated through the Maternal and Children's Health ward, including the head of the ward, were trained by investigator SM on the meaning of each of the questions in the survey and how to administer it, in consultation with a psychiatrist in Addis Ababa (see Acknowledgments). SM was present during all of the interviews conducted by the nurses to ensure they followed the proper protocol for administering the survey according to the WHO guidelines (Beusenberg et al., 1994). In order to minimize the impact of the power differential, SM sat together with the nurses to discuss the research about identification of mental illness and emphasized that the nurses were under no obligation to administer the survey. The head nurse, Sister Meseret, led survey implementation.

During postpartum visits, mothers were seated while their child received their immunization, and only the nurse, mother, and researcher were present. Following the child's immunization, nurses asked women if they were willing to answer 20 yes or no questions about their emotional wellness in the 30 days prior to their clinic visit. After obtaining verbal, informed consent, nurses administered the survey to the women as part of their postpartum checkup, including basic sociodemographic information at the beginning of the survey, to learn if they had experienced any psychological distress or postpartum depressive symptoms, and whether they were willing to discuss their emotions. Given that this was the first time that mental health was addressed at this hospital, we focused data collection on the SRQ and minimized other data collection. To combat social desirability bias, the same neutral language was used for every survey conducted, and the women's data was deidentified using participant codes that were linked to the women's names on a password-protected document on SM's Protected Health Information-compliant computer. Additionally, the nurses were in a healthcare setting that protects patient privacy. SM followed along using the English version of the survey as well as the script that the nurses were using to introduce the survey to make sure they were all following it accurately. SM observed and interjected when the nurses strayed from their instructions and training on administration of the survey to ensure consistency and minimize bias. To ensure survey quality and adherence to the questionnaire, these observations were recorded and discussed with the nurses in a debriefing session following the administration of each survey. In debriefing sessions, SM invited observations from the nurses on the survey process from the nurses.

A study in Butajira found that using a 4/5 cutoff score—meaning five or more affirmative answers—indicated presence of mental disturbance with 80% sensitivity and specificity (Hanlon et al., 2008). Using this study as reference, for all enrolled postpartum women we summed and reported positive (“Yes”) responses by question number and determined the prevalence of mental disturbance using a primary cutoff of 4/5, as those identified in the positive category using the 4/5 cutoff were noted in a previous study as in need of further psychiatric evaluation by a licensed professional (Rahman et al., 2013).

### Assessment of Mental Health Stigma

**Figure 1:**
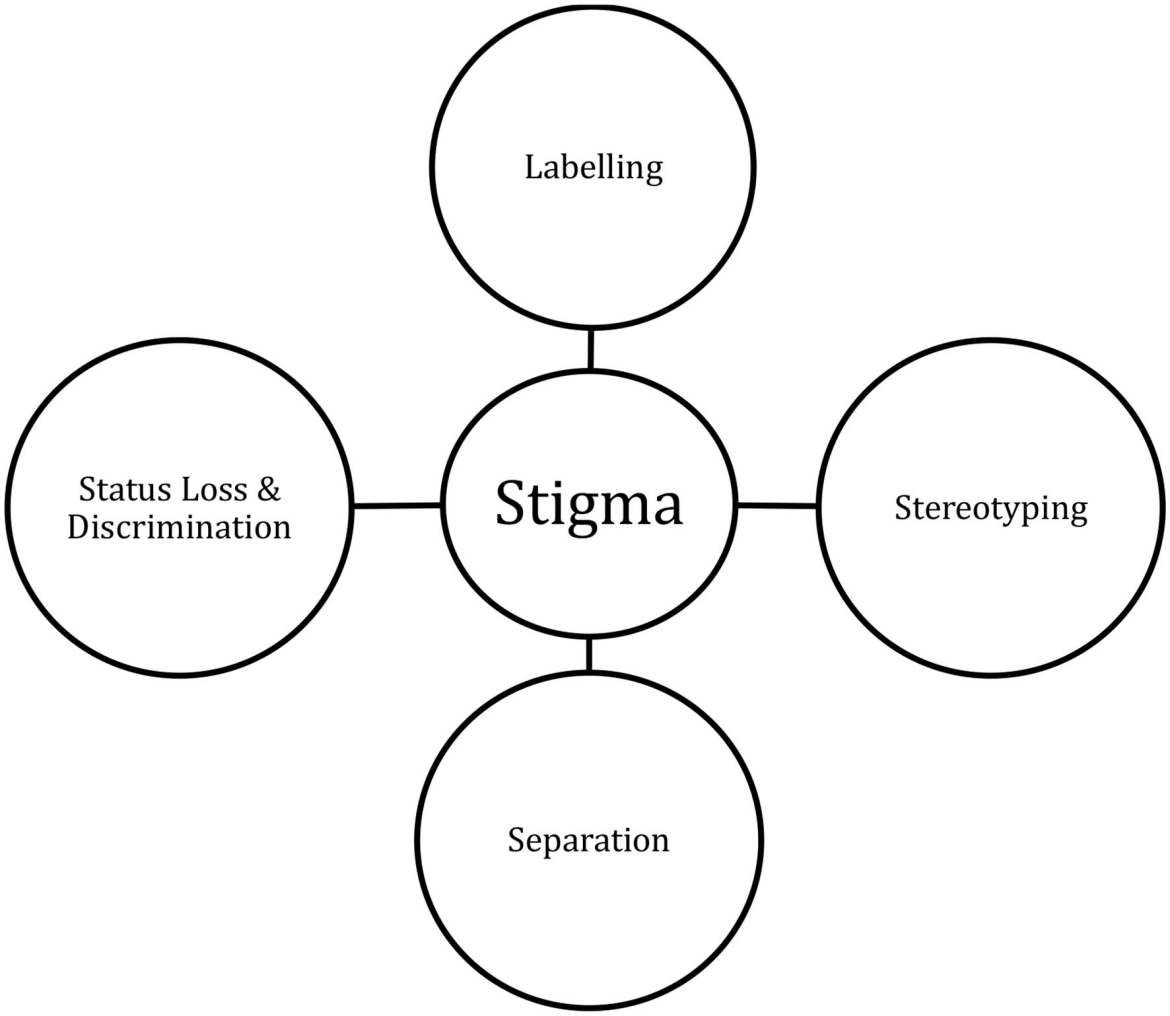
Conceptual framework for identification of stigma. Adapted from Link and Phelan (2001).

The presence of stigma, defined as a sign of disgrace or discredit which sets a person apart from others, was also assessed in the health care staff giving the survey, based on observations of survey administration by SM (Byrne, 2000). The main model used for observational data on stigma was adapted from Link and Phelan's Components of Stigma (2001) that describes converging components of: (1) labeling, (2) stereotyping, (3) separating, and (4) status loss and discrimination to identify stigma (Figure 1; Link and Phelan, 2001). During administration of the SRQ, we observed for these four signs from the health care staff. The nurses were not informed of the stigma assessment in order to reduce potential bias; however, as part of the informed consent process they were told that SM was conducting a research project on mental health. Observations on the presence of stigma on the part of the nurses were not discussed during survey administration debriefing sessions to enable ongoing assessment of its presence. Moreover, the rigorous nature of supervision to survey administration and regular discussions of observations of the researcher were designed to ensure that stigma did not significantly affect the survey results.

SM used a pre-determined framework adapted from Link and Phelan to record qualitative observations on the components of stigma, without use of prompts, using a printed framework guide for each postpartum woman. This involved four categories that were previously defined and behaviors that fell under one of those clearly defined categories were recorded (Figure 1). Labeling occurs when people distinguish and label human differences, so it was noted every time one of the health care staff denoted one of the women as different, or used a particular label, because of how she was acting or answering the survey. For example, labeling was observed when health care staff expressed the belief that their community and race (of females in particular) were mentally superior to those of the first world because they were “strong” and “never cry.” The second component of stereotyping occurs when “dominant cultural beliefs link labeled persons to undesirable characteristics—to negative stereotypes.” (Link and Phelan, 2001). This was recorded when the health care staff would assign a certain characteristic to women they had previously labeled as a result of their definition of Ethiopian culture. The third component of separating occurs when those who have been labeled are assigned to a specific, distinct category that achieves a separation of them from the person who assigns it to them, i.e., “us” vs. “them.” When health care staff made comments on distinct categorization of the women that had been labeled, this was also recorded. The fourth component of stigma relates to status loss and discrimination, a measure harder to achieve in the one-on-one interactions in the clinic; however, further research into the practices of the Ethiopian culture highlighted the link between the first three components and resulting status loss or discrimination (Reta et al., 2016). When each of these elements come together so that identifying those as different and constructing stereotypes to place them into categories leads to their exclusion from aspects of society as well as discrimination against them that adversely affects their access to social, economic, and political power, this is identified as stigmatization (Link and Phelan, 2001).

### Data Analysis

SPSS was used to quantify SRQ scores for all 20 questions across all respondents and to determine the proportion of respondents meeting the 4/5 cutoff for screen positivity for the presence of common mental disorders. For qualitative analysis of stigma, Link, and Phelan's framework was followed in order to interpret the observations collected.

### Ethics Approval and Consent to Participate

Ethical approval was received from the governing non-governmental organization of the hospital, Project Mercy, and the protocol was deemed exempt from human subjects review by the Stanford Institutional Review Board. All interviews were conducted during routine postpartum appointments after obtaining verbal informed consent; confidentiality and a comfortable environment were assured.

## Results

### Observations on Survey Administration

We surveyed 118 postpartum mothers in Project Mercy's Glenn C. Olsen Memorial Primary Hospital. Participants' median age was 30 years (interquartile range 22–34, full range 18–37); all but two were married, and they had an average of four children.

No women refused to participate in the survey, and all women answered all 20 questions. During survey administration women often laughed or vehemently denied aspects of mental health such as suicidal thoughts and crying. Respondents uniformly appeared reluctant to discuss their emotions.

### Presence of Postpartum Common Mental Disorders

Of the 118 women interviewed, 30.1% had experienced a loss of appetite and 24.8% had been feeling nervous, tense, or worried (Table 1). Overall, 18.6% answered yes to five or more of the 20 questions, indicating possible presence of common mental disorder using the 4/5 cut-off criteria. Two women (1.7%) admitted to having either considered or attempted suicide, for which they were referred to the psychiatric clinic in the neighboring city. Since only 2% of women interviewed were unmarried, we could not assess the prevalence of mental disorders in unmarried vs. married women.

**Table 1:** Proportions of women who answered Self Reporting Questionnaire questions positively.

**SRQ survey questions**	**% yes**
Experienced loss of appetite	30.1%
Been nervous, tense, or worried	24.8%
Experienced poor digestion	18.6%
Had headaches quite often	17.7%
Had problems thinking clearly	17.7%
Daily activities suffered	15.9%
Felt tired all the time	15.0%
Problems with sleep	15.0%
Felt tired easily	15.0%
Frightened easily	10.6%
Had uncomfortable feelings in stomach	9.7%
Found it difficult to make decisions	6.2%
Found it difficult to enjoy daily activities	5.3%
Often lost interest in things	5.3%
Cried more than usual	3.5%
Often experienced shaking of hands	3.5%
Felt unable to play a useful part in life	3.5%
Thought of ending life	1.8%
Generally felt unhappy	1.8%
Felt worthless	0.9%

### Stigma

Labeling was observed in the nurses' denial of the existence of depression in the community. The nurses repeatedly suggested, following survey distribution, “That is not a problem in our country.” Nurses maintained that the women of the community were strong and never cried or experienced sadness. One nurse commented: “We are strong as Ethiopians. These women do not have time to be sad, they are carrying hay bales to market on their backs. They are strong, not sad.” There were multiple comments indicative of stereotyping, such as “those who experience this sadness, for example those in the first world, are religiously weak or not in favor with God.” Another nurse noted: “Because in America life is easier, people find reasons to give themself difficulty and create sadness.” Crying was associated with weakness and religious sin, which strongly correlates to the strong religious culture of Ethiopia. During administration of the survey, when a woman answered “yes” to the question about crying, the nurses commonly asked her “are you sure?” and would laugh and appear to try to convince her it was important to be strong. As this behavior deviated from how the nurses were trained, it was immediately pointed out by the researcher and the behavior subsided yet persisted over the course of the study. In instances in which these women remained steadfast in their answer, as well as in the case of the two women who admitted to attempting suicide, following the survey in private, the head nurse suggested that they were not a part of the strong community and were not the same as everyone else. This served to fulfill the third aspect of stigma as described by Link and Phelan, distinctly categorizing these women as a result of their answer so as to achieve an “us” vs. “them”. Mental illness was denied as an explanation for symptoms, and over-exhaustion and headaches (some of the indicators in the survey) were attributed to malnutrition by the nurses. The majority of comments made and discussions resulted in a grouping of “mental illness” with “weakness” and “Ethiopian” as “strength.” Further discussions with and observations of the community members and staff at Project Mercy confirmed that those with psychosis were often not allowed to take part in community activities, are excluded from employment and educational opportunities, and are kept or chained up in their homes without proper access to the medical attention they need, which fulfills the fourth characteristic of status loss and discrimination (Link and Phelan, 2001).

## Discussion

The main objectives of this research were to determine the prevalence of postpartum common mental disorders in the rural community of Yetebon, and to assess whether stigma existed in the health care staff and community that could potentially bias the identification of women with psychological distress. The data supports that mental disorders are common among postpartum women in the Yetebon community. Using the 4/5 cut-off score, the prevalence of possible cases was 18%. However, the sample size was limited. Further studies are needed to define the prevalence of common mental disorders in postpartum women in Ethiopia. In particular, further research is needed on perceptions of mental illness by community members, as this could impact the way in which the SRQ survey questions are understood and answered, thus affecting the prevalence reported (Monteiro and Balogun, 2013a; Abbay et al., 2018). The criteria for stigma created by Link and Phelan were met through observation and conversations with the health care staff and community members (Link and Phelan, 2001). Both the postpartum women as well as the health care staff equated their identity with not having mental illness, a perception that could potentially hinder their ability to determine or admit when an individual had mental disturbance and could impact the provision of mental health services to the postpartum women in follow-up care.

Link and Phelan concluded that the existence of stigma can be confirmed when “stereotyping, separation, status loss and discrimination co-occur,” each of which was observed over the course of this 9-month study (Link and Phelan, 2001). Observing elements of each characteristic of stigma suggests that there was stigma among the health care staff and likely in the community as well. This stigma may lead to both direct and indirect effects on the health and well-being of the Yetebon community, since both maternal (direct effect) and children's (indirect effect) health could be impacted by this lack of recognition and available mental health education and services for health care staff and postpartum women. Based on a study done in Butajira, about 20 km away, children whose mothers experienced common mental disorders and expressed symptoms were more likely to drop out of school or be absent during preschool and early elementary school (Mekonnen et al., 2018). Maternal depression has also been shown to potentially negatively affect children's Intelligence Quotient (Wu et al., 2018). Various studies, however, have documented that early identification, intervention, and treatment of maternal depression had positive impacts on the women (Baron et al., 2016), and the resources provided were important in allowing them to not only manage their own symptoms but also to engage with their children to enable them to reach their full potential (Mekonnen et al., 2018; Wu et al., 2018).

Since few to no nurses in the Glenn C. Olsen Memorial Primary Hospital have received any formal mental health pre-service education or in-service training, our data showing the presence of common mental disorders in nearly one-in-five postpartum women, as well as stigma among healthcare staff leads us to believe that both mothers' and children's health outcomes could be improved with implementation of mental health support. A key approach to addressing the stigma observed will be to improve understanding of community perceptions of mental illness as well as health care workers' knowledge of mental health through proper education and government investment in health extension worker and nursing programs. There has been a push from the global health field to integrate maternal mental health care into maternal and child health services (Baron et al., 2016). Ethiopia's mental health strategy created by the government has now made it a requirement for primary hospitals to implement mental health services within their healthcare system (i.e., placement of a psychiatric physician). This is not strictly mandated or monitored, but nonetheless provides an important incentive for discussion and actions to address maternal mental health. The government has called for use of health extension workers “for promotion and prevention activities to increase awareness, reduce stigma, and increase use of mental health services,” (Federal Democratic Republic of Ethiopia, Ministry of Health (MOH), 2012) something we have yet to see in the community of Yetebon or hear of in other rural communities in the Gurage Region.

There were various limitations of this study that indicate a need for further research. Firstly, we only surveyed women who came to the hospital for routine vaccinations or postnatal care so out of the 400 women who gave birth in the hospital throughout the course of the study ~30% were surveyed. It is possible that unmarried women were ashamed to come in or those who were too ill or depressed to come in may have not had the opportunity to be surveyed, which could explain a lower prevalence than the one found in other studies. In future studies, the surveyors could go house-to-house and ensure that all women who gave birth in the geographic area of Yetebon are given the opportunity to complete the survey. Additionally, those who administered the surveys did not appear to be impartial, and therefore the manner in which they asked the survey questions and potentially deviated from the script may have introduced reporting bias. We attempted to minimize this bias through the researcher's supervision of the process and debriefings, but acknowledge that it could not be completely addressed. This reporting bias coupled with a social desirability bias also likely existed in the women from the community who presented for postpartum care. Thus, our estimate of the prevalence of common mental disorders in this community may be an under-estimate, and requires further investigation. Another limitation was the methodology; the cross-sectional nature of the study with regard to individual participants did not allow for drawing temporal relationships between the variables. Also, since this was the first attempt to assess the prevalence of mental illness in this hospital and community, we minimized the questions asked. However, as a result, we lacked epidemiological and demographic covariates for use in regression analysis. In subsequent research it will be important to collect additional data and seek to establish risk profiles for common mental disorders. Continued discussion of the findings and implications, and additional qualitative research to highlight and amplify the voices of Ethiopians (mothers and health workers) is vital in helping to increase our understanding of local perceptions and management of mental illness and the socio-cultural context that inform interactions between health care workers and community members to assess the presence of, and treatment approaches for mental illness. Only then can accurate identification, diagnosis and management of the range of mental illnesses be effectively administered.

In addition to further formative research to understand community perceptions of mental illness, an important step is to facilitate educational opportunities for health care staff, not only so future research can be done with decreased bias and stigma, but also so that when women are found to have mental disorders, stigma, on the one hand, and availability of services on the other hand, can be taken into account in referring women to providers who are acceptable, able and qualified to diagnose and treat them.

### Conclusion

This research demonstrated the common presence of postpartum mental illness in the community as well as a strong prevalence of stigma against acknowledging or diagnosing mental illness. Stigma was observed in the maternal health department where women are expected to be strong before, during and after giving birth to children, regardless of emotional or physical health. There is an urgent need for healthcare staff to receive additional education and supervision in the identification and referral of postpartum women with common mental disorders. The Ethiopian Government has a national mental health strategy, a step toward the provision of the funds and support necessary to improve mental healthcare access throughout the country (Federal Democratic Republic of Ethiopia, Ministry of Health (MOH), 2012). By presenting this research on the prevalence of common mental disorders in postpartum women and the presence of stigma among the healthcare staff, we hope to stimulate increased availability of properly trained and supervised health extension workers in the town of Butajira and the surrounding woredas and kebeles, and the availability of medical professionals to treat mental illness with compassion, understanding, and skill.

## Data Availability Statement

The original contributions presented in the study are included in the article/supplementary material, further inquiries can be directed to the corresponding author.

## Ethics Statement

The study protocol was reviewed and approved by the governing non-governmental organization of the hospital, Project Mercy, and the protocol was deemed exempt from human subjects review by the Stanford Institutional Review Board. All interviews were conducted during routine postpartum appointments after obtaining verbal informed consent; confidentiality and a comfortable environment were assured.

## Author Contributions

GD and SM conceived the study and were responsible for the study design, interpretation of results, and writing of the paper. BD arranged access to the postpartum ward. MA performed and SM supervised data collection. SM conducted data analysis. BD provided critical review of the paper, and all authors approved the paper for publication.

## Conflict of Interest

MA was an employee, BD was President, and GD was Chair of the Board of Directors of Project Mercy at the time the study was conducted. The remaining author declares that the research was conducted in the absence of any commercial or financial relationships that could be construed as a potential conflict of interest.

## Abbreviations

LMICs: Low- and middle-income countries
SRQ: Self Reporting Questionnaire
WHO: World Health Organization.
